# Alterations in Brain Network Topology and Structural-Functional Connectome Coupling Relate to Cognitive Impairment

**DOI:** 10.3389/fnagi.2018.00404

**Published:** 2018-12-13

**Authors:** Juan Wang, Reza Khosrowabadi, Kwun Kei Ng, Zhaoping Hong, Joanna Su Xian Chong, Yijun Wang, Chun-Yin Chen, Saima Hilal, Narayanaswamy Venketasubramanian, Tien Yin Wong, Christopher Li-Hsian Chen, Mohammad Kamran Ikram, Juan Zhou

**Affiliations:** ^1^Center for Cognitive Neuroscience, Neuroscience and Behavioral Disorders Program, Duke-National University of Singapore Medical School, Singapore, Singapore; ^2^Institute for Cognitive and Brain Sciences, Shahid Beheshti University, Tehran, Iran; ^3^Department of Pharmacology, National University of Singapore, Singapore, Singapore; ^4^Neuroscience Clinic, Raffles Hospital, Singapore, Singapore; ^5^Memory Aging & Cognition Centre, National University Health System, Singapore, Singapore; ^6^Singapore National Eye Centre, Singapore Eye Research Institute, Singapore, Singapore; ^7^Department of Neurology, Brain Center Rudolf Magnus, University Medical Center Utrecht, Utrecht, Netherlands; ^8^Clinical Imaging Research Centre, The Agency for Science, Technology and Research-National University of Singapore, Singapore, Singapore

**Keywords:** cognitive impairment no dementia, functional connectome, structural connectome, structural-functional coupling, task-free fMRI, diffusion tensor imaging (DTI)

## Abstract

According to the network-based neurodegeneration hypothesis, neurodegenerative diseases target specific large-scale neural networks, such as the default mode network, and may propagate along the structural and functional connections within and between these brain networks. Cognitive impairment no dementia (CIND) represents an early prodromal stage but few studies have examined brain topological changes within and between brain structural and functional networks. To this end, we studied the structural networks [diffusion magnetic resonance imaging (MRI)] and functional networks (task-free functional MRI) in CIND (61 mild, 56 moderate) and healthy older adults (97 controls). Structurally, compared with controls, moderate CIND had lower global efficiency, and lower nodal centrality and nodal efficiency in the thalamus, somatomotor network, and higher-order cognitive networks. Mild CIND only had higher nodal degree centrality in dorsal parietal regions. Functional differences were more subtle, with both CIND groups showing lower nodal centrality and efficiency in temporal and somatomotor regions. Importantly, CIND generally had higher structural-functional connectome correlation than controls. The higher structural-functional topological similarity was undesirable as higher correlation was associated with poorer verbal memory, executive function, and visuoconstruction. Our findings highlighted the distinct and progressive changes in brain structural-functional networks at the prodromal stage of neurodegenerative diseases.

## Introduction

Neurodegenerative diseases target large-scale neural networks (Braak and Braak, 1991; Seeley et al., 2009; Zhou et al., 2012; Wang et al., 2015b). Networks, such as the default mode network, dorsal attention network, and the salience network have been shown to be “epicenters” of different dementia subtypes, with the differential involvement of these networks closely tied with the symptomatic differences across subtypes, and can be contrasted with normal age-related brain network degradation (Zhou and Seeley, 2014; Chong et al., 2017; Chhatwal et al., 2018). These observations have served as the solid foundation of the network-based selective vulnerability hypothesis (Seeley et al., 2009; Greicius and Kimmel, 2012). Transneuronal spread model is one possible mechanism in which disease agents, such as tau and amyloid would transmit through neural pathways characterized by functional and/or structural relationship instead of spatial proximity between brain regions (Greicius and Kimmel, 2012; Raj et al., 2012; Zhou et al., 2012). Human brain neural networks have been directly probed by structural connectivity derived from diffusion tensor imaging (DTI) and functional connectivity derived from task-free or resting-state functional magnetic resonance imaging (fMRI) (Wang et al., 2015b; Teipel et al., 2016; Zhou et al., 2017). Converging neuroimaging evidence from our group and others have shown syndrome-specific structural and functional network disruptions in Alzheimer's disease (AD) and other types of neurodegenerative disorders (Greicius et al., 2004; He et al., 2009; Zhou et al., 2010; Zhou and Seeley, 2014), highlighting the valuable insights of understanding these disorders with a network-based approach. Applied on both whole-brain structural and functional connectivity data (connectomes), graph theoretical measures characterize complex brain neural network topology and quantify network integration, modularity, or efficient communications using both nodal and global indices (Bullmore and Sporns, 2009; Rubinov and Sporns, 2010), and has been an exellent tool for probing brain structural and functional networks perturbed by neurodegnerative disorders (Stam, 2014).

Considerable effort has been devoted to better understand the changes in neural network topology and its associations with symptoms manifestation in prodromal stages of dementia, such as participants with mild cognitive impairment (MCI) and cognitive impairment no dementia (CIND) (Hughes et al., 2011; Karantzoulis and Galvin, 2011). In prodromal and clinical AD, the structural connectome (SC) exhibits altered topological network metrics, such as increased shortest path lengths and decreased local and global efficiency (Lo et al., 2010; Bai et al., 2012b; Shao et al., 2012). In parallel, similar functional connectomic disruptions have been reported in prodromal AD (Greicius et al., 2004; Rombouts et al., 2005; Sorg et al., 2007) and asymptomatic individuals at risk of AD (Filippini et al., 2009). Regional (or nodal) topological changes, in particularly the default mode network, are also frequently reported. For instance, the brain structural and functional hubs, characterized by high centrality or the participant coefficient and distributed across many brain networks (e.g., posterior cingulate cortex, precuneus, medial prefrontal gyrus, and temporal gyrus), were reported to lose their hub-like character in AD and MCI compared to healthy controls (Buckner et al., 2009; Brier et al., 2014) and was associated with cognitive deficits (Reijmer et al., 2013; Dicks et al., 2018). Nevertheless, few studies have compared structural and functional network patterns at different stages of MCI or CIND (Wang et al., 2011, 2012). A recent study in MCI patients revealed that the intra- and inter-network functional connectivity disruptions were more profound in late MCI compared to the early MCI patients (Zhan et al., 2016). Similar to the MCI classification, work on the topological changes in both structural and functional connectome underlying CIND cognitive decline remains largely unknown (Zhu et al., 2014).

Recent work has found that structural connectivity could shape and impose constraints on the pattern of functional connectivity in health (Hagmann et al., 2010; Goni et al., 2014). Both human and animal studies have shown that the functional connectome (FC) at the unconscious state is more similar to SC (Barttfeld et al., 2015) and time spent in the more SC-like state is associated with poorer vigilance task performance (Wang et al., 2016). On the other hand, FC patterns can also in turn reflect underlying structural architecture in health and disease (Greicius et al., 2009; Wang F. et al., 2009; Zhang et al., 2014; Vecchio et al., 2015). More importantly, the structural-functional relationships of large-scale brain networks may be disrupted in disease (Hagmann et al., 2010; Wang et al., 2015a) and the two might interact to relate to cognitive deficits (Qiu et al., 2016). Yet, brain network SC-FC coupling changes at the system-level in mild and moderate CIND individuals needs to be established.

To this end, we compared the brain SC and FC in individuals with mild CIND (two or fewer domain deficits), moderate CIND (multi-domain deficits), and no cognitive impairment (NCI) using graph theoretical approach (Rubinov and Sporns, 2010). We hypothesized that moderate CIND rather than mild CIND would exhibit greater deterioration in SC and FC, such as reduced global and nodal efficiency, compared to NCI. Moreover, given previous findings on the close association of high SC-FC similarity with reduced consciousness and lower vigilance, we expected that CIND (moderate rather than mild) would have higher SC-FC coupling than NCI and such changes would be associated with cognitive impairment.

## Materials and Methods

### Participants

Participants were drawn from the ongoing Epidemiology of Dementia in Singapore study (Hilal et al., 2013), which is part of the Singapore Epidemiology of Eye Disease study (Huang et al., 2015). The present analysis was restricted to the Chinese component of the Epidemiology of Dementia in Singapore study, in which neuroimaging data were available. Out of 261 Chinese participants, we studied 56 participants with moderate CIND, 61 participants with mild CIND, and 97 healthy older adults with NCI (Table 1). Each participant completed all the clinical and neuropsychological evaluation and passed the quality control of neuroimaging data (see “network construction” for details). The study was conducted in accordance with the Declaration of Helsinki and approved by the Singapore Eye Research Institute and the National Healthcare Group Domain Specific Review Board. Written informed consent was obtained from each participant prior to recruitment into the study.

**Table 1:** Subject demographics and clinical characteristics.

	**NCI (*n* = 97)**	**Mild CIND (*n* = 61)**	**Moderate CIND (*n* = 56)**	***p*-value**
Age (years)	60–86 (67.0 ± 4.7)	60–84 (71.0 ± 6.4)^n^	62–85 (74.0 ± 5.3)^nm^	< 0.0001^***^^T^
Gender (F/M)	42/55	33/28	40/16^n^	< 0.05^*^
Handedness (R/L)	94/3	61/0	54/2	0.357
CDR-SB	0–2.5 (0.08 ± 0.3)	0–1 (0.24 ± 0.35)^n^	0–3.5 (0.86 ± 0.81)^nm^	< 0.001^**^^T^
MMSE	21–30 (26.7 ± 1.7)	17–29 (24.5 ± 2.5)^n^	14–28 (21.0 ± 3.4)^nm^	< 0.0001^***^^T^
MoCA	15–30 (24.2 ± 2.9)	9–29 (20.3 ± 3.5)^n^	6–24 (15.7 ± 4.4)^nm^	< 0.0001^***^^T^
Executive	−1.11–1.50 (0.51 ± 0.65)	−2.24–1.50 (0.12 ± 0.80)^n^	−3.36–0.75 (−1.02 ± 0.95)^nm^	< 0.0001^***^^T^
Attention	−1.30–2.61 (0.56 ± 0.65)	−1.75–1.39 (0.03 ± 0.72)^n^	−3.80–0.95 (−1.00 ± 1.00)^nm^	< 0.0001^***^
Language	−1.81–2.22 (0.62 ± 0.71)	−1.89–1.70 (−0.04 ± 0.72)^n^	−2.91–0.46 (−1.03 ± 0.80)^nm^	< 0.0001^***^
Verbal memory	−0.59–2.58 (0.71 ± 0.68)	−1.34–2.27 (−0.28 ± 0.79)^n^	−2.48–1.33 (−0.92 ± 0.73)^nm^	< 0.0001^***^
Visual memory	−0.68–1.98 (0.75 ± 0.67)	−2.29–1.52 (−0.23 ± 0.70)^n^	−2.79–0.27 (−1.04 ± 0.65)^nm^	< 0.0001^***^
Visuoconstruction	−0.73–2.18 (0.68 ± 0.53)	−2.31–1.71 (−0.02 ± 0.73)^n^	−2.67–1.00 (−1.15 ± 0.80)^nm^	< 0.0001^***^^T^
Visuomotor speed	−0.51–2.18 (0.72 ± 0.57)	−1.69–1.38 (−0.13 ± 0.76)^n^	−2.28–0.96 (1.13 ± 0.68)^nm^	< 0.0001^***^^T^

*Data are presented as range (mean ± standard deviation). Superscript letters indicate the mean index of the representing group was significantly different from “n”–no cognitive impairment (NCI) or “m”–mild CIND based on two-sample t-test or Pearson's χ^2^ test (p < 0.05). NCI, no cognitive impairment; CIND, cognitive impairment no dementia; CDR-SB, Clinical Dementia Rating Scale Sum of Boxes; MMSE, Mini Mental State Examination; MoCA, Montreal Cognitive Assessment; F, female; M, male; L, left; R, right; T, Tamhane's T2*.

* *p < 0.05*,

** *p < 0.001*,

*** *p < 0.0001 for omnibus ANOVA and χ^2^ tests*.

### Neuropsychological Assessments and Diagnoses

Trained research psychologists administered brief cognitive screening tests, the Clinical Dementia Rating Scale (CDR) (Morris, 1993; Greve and Fischl, 2009), the Mini-Mental State Examination (MMSE) (Folstein et al., 1975), the Montreal Cognitive Assessment (MoCA) (Nasreddine et al., 2005), the informant questionnaire on cognitive decline (Jorm, 2004), and a formal neuropsychological battery locally validated for older Singaporeans (Yeo et al., 1997). For the five non-memory domains, (1) executive function was assessed with frontal assessment battery (Dubois et al., 2000) and maze task (Porteus, 1959); (2) attention was assessed with digit span, visual memory span (Wechsler, 1997) and auditory detection tests (Lewis and Rennick, 1979); (3) language was assessed with the Boston naming test (Mack et al., 1992) and verbal fluency (Isaacs and Kennie, 1973); (4) visuomotor speed was assessed with the symbol digit modality test (Smith, 1973) and digit cancellation (Diller et al., 1974); (5) visuoconstruction was assessed with Weschler memory scale revised visual reproduction copy task (Wechsler, 1997), clock drawing (Sunderland et al., 1989) and Weschler adult intelligence scale-revised subtest of block design (Wechsler, 1981). For the two memory domains, (1) verbal memory was assessed with word list recall (Sahdevan et al., 1997) and story recall; (2) visual memory was assessed with picture recall and Weschler memory scale-revised visual reproduction (Wechsler, 1997). The assessment was administered in the participant's habitual language. Domain-specific z-scores were obtained by averaging the z-scores of the subtests belonging to that domain.

Diagnoses of cognitive impairment and dementia were made at weekly consensus meetings in which study clinicians, neuropsychologists, clinical research fellows, research coordinators, and research assistants reviewed clinical features, blood investigations, psychometrics, and neuroimaging data. Failure in a test was defined using education adjusted cut-off values of 1.5 standard deviations below the established normal means on individual tests (Hilal et al., 2013). Failure in half of the tests in a domain constituted failure in that domain. Following our previous work (Hilal et al., 2013; Chong et al., 2017), CIND was defined as impairment in at least one domain of the neuropsychological test battery. To refine cognitive impairment severity, mild CIND was diagnosed when two or fewer domains were impaired and moderate CIND as impairment in more than two domains.

### Imaging Acquisition

All structural and functional images were collected using a 3T Siemens Allegra system (Siemens, Erlangen, Germany). Each participant underwent a T1-weighted structural MRI, a task-free fMRI and a DTI scan in the same session. High-resolution T1-weighted structural MRI was acquired using MPRAGE (magnetization-prepared rapid gradient echo) sequence (192 continuous sagittal slices, TR/TE/TI = 2,300/1.9/900 ms, flip angle = 9°, FOV = 256 × 256 mm^2^, matrix = 56 × 256, isotropic voxel size = 1.0 × 1.0 × 1.0 mm^3^, bandwidth = 240 Hz/pixel). DTI was obtained using a single-shot, echo-planar imaging (EPI) sequence (61 non-collinear diffusion gradient directions at b = 1,150 s/mm^2^, seven volumes of b = 0 s/mm^2^, TR/TE = 6,800/85 ms, FOV = 256 × 256 mm^2^, matrix = 84 × 84, 48 contiguous slices, and slice thickness = 3.0 mm). A 5-min task-free fMRI were acquired using a single-shot EPI sequence (TR/TE = 2,300/25 ms, flip angle = 90°, FOV = 192 × 192 mm, matrix = 64 × 64, 48 contiguous axial slices, and voxel size = 3.0 × 3.0 × 3.0 mm^3^).

### Construction of Brain Networks

#### Brain Anatomical Parcellation

**Figure 1:**
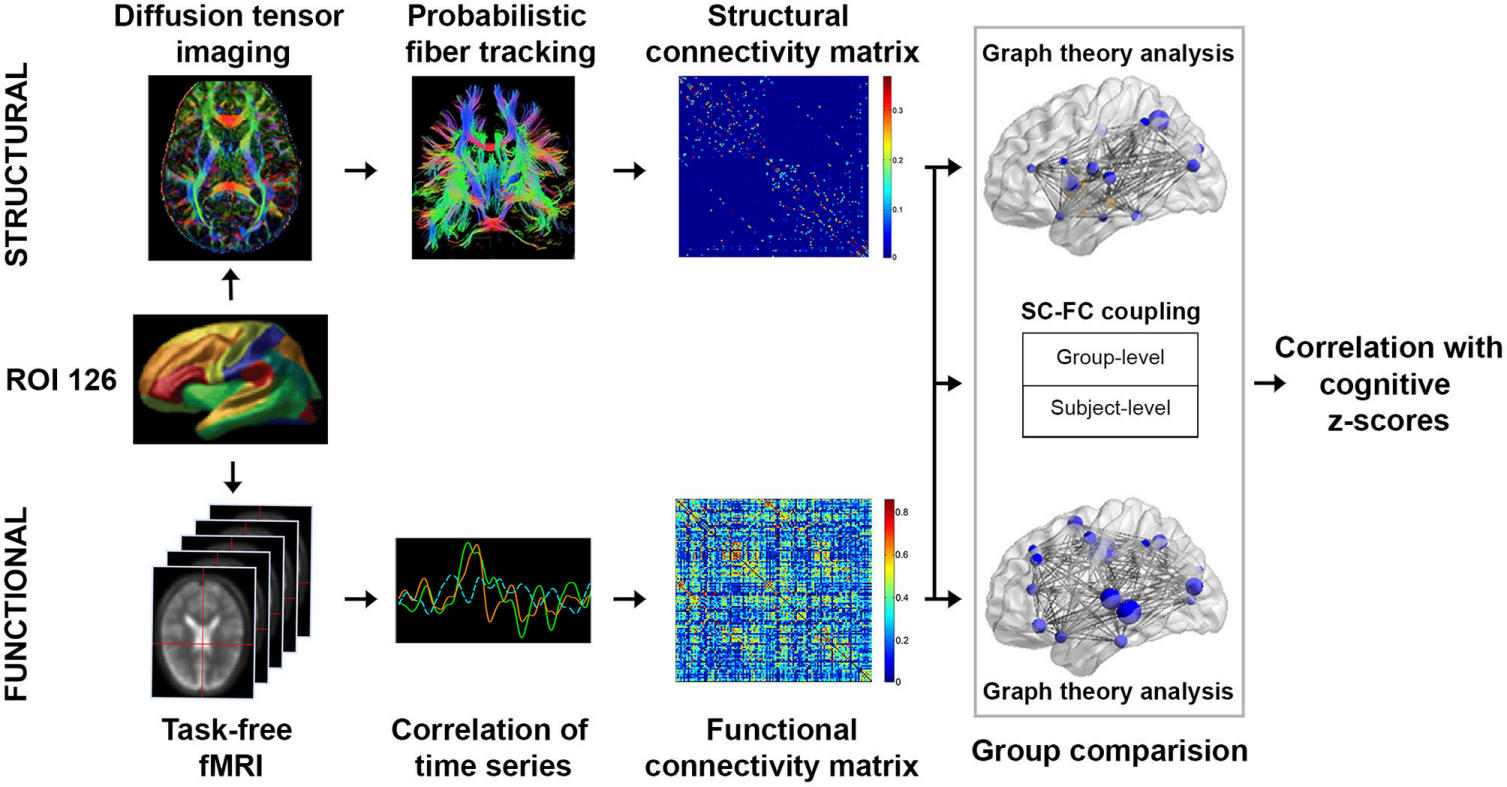
Study design schematic. We studied the network topological changes in patients with mild cognitive impairment no dementia (CIND) and moderate CIND compared to healthy controls. Subject-level structural connectome (SC) was derived from diffusion MRI data using probabilistic fiber tracking based on 126 regions of interests (see Methods for details). Subject-level functional connectome (FC) was derived from task-free fMRI data based on pairwise Pearson's correlations between the 126 regions of interests. Graph theoretical global-wise and nodal-wise metrics were computed for both connectomes. Furthermore, SC-FC coupling was evaluated as the Pearson's correlation between the structural matrices and functional matrices both at the individual and group levels. Alterations in structural and functional network topology metrics as well as SC-FC coupling measures were compared across groups and subsequently associated with cognitive performance.

To characterize brain functional architecture, we constructed SC and FC based on a predefined set of 126 regions of interest (ROIs), which included 114 cortical ROIs covering 17 functionally parcellated networks derived from resting state fMRI-based FC (Yeo et al., 2011), and 12 subcortical regions from the automatic anatomical labeling (AAL) template (Tzourio-Mazoyer et al., 2002) (Figure 1). See Wang and colleagues for an exemplary application of the same approach (Wang et al., 2016).

#### Construction of Structural Connectome

Diffusion related data was preprocessed using FSL (http://www.fmrib.ox.ac.uk/fsl) following an approach described previously (Cortese et al., 2013). T1-weighted structural images were deobliqued prior to reorientation to the diffusion space and then skull stripped using the Brain Extraction Tool (Smith, 2002). DTI data were corrected for Eddy current distortion and head movement through affine registration of diffusion-weighted images to the first b = 0 volume. Data were discarded if the maximum displacement relative to this volume was more than 3 mm. Diffusion gradients were rotated to improve consistency with the motion parameters. Fractional Anisotropy images were created by fitting a diffusion tensor model to the diffusion data at each voxel. The probabilistic distribution of diffusion parameters at each voxel was built up by the Bayesian estimation of diffusion parameters (bedpostx) (Behrens et al., 2003, 2007).

To construct the SC for each participant, (1) each individual's preprocessed diffusion image was co-registered to the participant's high-resolution T1 structural image using Boundary-Based-Registration (BBR) (Greve and Fischl, 2009). (2) T1 structural image was non-linearly registered to MNI space (FNIRT). (3) The derived transformation parameters were inversed and applied on the ROI templates to register back into diffusion native space. ROI label intensity was kept by using nearest neighbor interpolation. (4) Probabilistic fiber tracking was performed in the parcellated diffusion native space using PANDA (Gong et al., 2009; Cui et al., 2013) to derive the probability connectivity matrix. (5) We assigned the connection probability between node *i* and *j, P*_*ij*_, as the mean of the connectivity probabilities from node *i* to *j* and from node *j* to *i* (Gong et al., 2009). (6) To account for different network costs in different subjects, the final weight for each pair of nodes, *w*_*ij*_, in the SC was obtained by normalizing *P*_*ij*_:

$$\begin{array}{l} w_{ij}=\frac{\log\left(P_{ij}\right)-\min{\left\{\log\left(P_{ij}\right)\right\}}_{1\leq i\neq j\leq N}}{\max{\left\{\log\left(P_{ij}\right)\right\}}_{1\leq i\neq j\leq N}-\min{\left\{\log\left(P_{ij}\right)\right\}}_{1\leq i\neq j\leq N}}. \end{array}$$

where *N* denotes the number of nodes (*N* = 126).

#### Construction of Functional Connectome

Task-free fMRI data was preprocessed using FSL (Smith et al., 2004; Woolrich et al., 2009; Jenkinson et al., 2012) and AFNI (Cox, 1996). Steps included (1) dropping the first 5 volumes, (2) slice time correction to the first slice, (3) deobliquing images prior to reorientation, (4) spatial realignment to the first volume, (5) band-pass filtering between 0.009 and 0.1 Hz, (6) spatial smoothing using a Gaussian filter of 6 mm FWHM, (7) detrending, (8) coregisteration to participants' high-resolution T1 structural images and then to FSL MNI152 standard template using linear (FLIRT) and non-linear (FNIRT) transformations, and (9) regressing out confounds of motion (six parameters), white matter, and cerebral spinal fluid. Participants with absolute motion exceeding 3 mm were discarded from analysis.

To construct the FC for each participant, (1) the functional connectivity (edges) between every pair of ROIs were estimated by computing the temporal correlations between the task-free fMRI blood-oxygen-level dependent (BOLD) signals (Friston et al., 1993) of the pairs. (2) Representative time series in each ROI were obtained by averaging the task-free fMRI time series across all voxels in the ROI. (3) A symmetric unsigned, undirected, and weighted functional connectivity network was then constructed by computing the absolute value of the Pearson correlations between the time series of every ROI pair, *Z*_*ij*_ = |*r*_*ij*_|, where *r*_*ij*_ is the Pearson correlation coefficient for nodes *i* and *j*. The main diagonal elements, i.e., self-connections, were set to zero.

### Graph Theoretical Analysis

Graph theoretical metrics were computed using the Brain Connectivity Toolbox (Rubinov and Sporns, 2010). We conducted the graph theoretical analysis over a range of cost thresholds (see section Network Thresholding and Topological Measures; Watts and Strogatz, 1998; Achard and Bullmore, 2007; He et al., 2008). Past studies have suggested that human brain structural and functional networks exhibit high local efficiency and global efficiency in information communication, accompanied with low wiring costs (i.e., sparse connections) (Latora and Marchiori, 2001). This “small-world” configuration is likely as a result of natural selection under the pressure of a cost-efficiency balance (Liao et al., 2017). Therefore, when choosing the range of cost, we adopted the commonly used method of ensuring small-world configuration of brain networks (Bassett et al., 2006; Zhang J. et al., 2011; Meng et al., 2014; Lu et al., 2017). The topological organizations of these networks were then characterized using global-wise metrics, namely local and global efficiency, and nodal-wise metrics, namely nodal degree centrality and nodal efficiency. These metrics were computed for both SC and FC graphs at each level of network costs, and then integrated across the cost range, resulting in one composite measure for each metric.

#### Small-world Brain Networks

A small-world network is highly segregated and integrated (Humphries et al., 2006). It has similar characteristic path length as a random network but more clustered (Watts and Strogatz, 1998; Pievani et al., 2011). It is defined as

$$\begin{array}{l} SW^{w}=\frac{C_{net}^{w}/C_{rand}^{w}}{L_{net}^{w}/L_{rand}^{w}}, \end{array}$$

Where $C_{net}^{w}$ and $C_{rand}^{w}$ are weighted clustering coefficients, and $L_{net}^{w}$ and $L_{rand}^{w}$ are the weighted characteristic path lengths of the respective tested network and a random network (see Supplementary Methods for details). Typically, a small-world network should have *SW*^*w*^>1 (Achard et al., 2006; He et al., 2007). To examine the small-worldness for each network, we generated 100 random networks with the same number of nodes, edges and degree distributions as the real network (Maslov and Sneppen, 2002). $C_{rand}^{w}$ and $L_{rand}^{w}$ were evaluated as the mean of weighted clustering coefficients and weighted characteristic path lengths based on the set of random networks.

#### Global-Wise Metrics: Global and Local Efficiency

The brain networks have economical small-world properties, supporting the massively parallel information processing, i.e., the nodes in the brain network sends information simultaneously by its edges (Latora and Marchiori, 2001; Achard and Bullmore, 2007). The global efficiency *E*_*glob*_ is a measure of the capacity for parallel information transfer in the network. It is defined as the inverse of the harmonic mean of shortest path length between each pair of nodes (Latora and Marchiori, 2001; Rubinov and Sporns, 2010):

$$\begin{array}{l} E_{glob}\left(G\right)=\frac{1}{\left(N-1\right)}\underset{1\leq i,j\leq N,i\neq j}{\sum }\frac{1}{L_{ij}}, \end{array}$$

where *G* represents the weighted brain network, *N* denotes the number of nodes and *L*_*ij*_ denotes the shortest path length between node *i* and *j*.

The local efficiency measures the capability of the network regarding information transmission at the local level (Latora and Marchiori, 2001):

$$\begin{array}{l} E_{loc}\left(G\right)=\frac{1}{N}\overset{N}{\underset{i=1}{\sum }}E_{glob}\left(G_{i}\right), \end{array}$$

where *G*_*i*_ is the neighborhood subgraph of the node *i*.

#### Nodal-Wise Metrics: Nodal Degree Centrality and Nodal Efficiency

The weighted nodal degree centrality presents the sum of the weights of all edges that are directly linked to a node. For a node *i*, nodal degree centrality is defined as

$$\begin{array}{l} D_{i}=\overset{N}{\underset{j=1}{\sum }}w_{ij}, \end{array}$$

where *w*_*ij*_ denotes the edge weight between node *i* and node *j*.

The nodal efficiency measures the ability of a node to propagate information with the other nodes in the network (Latora and Marchiori, 2001; Rubinov and Sporns, 2010):

$$\begin{array}{l} E_{i}=\frac{1}{\left(N-1\right)}\overset{N}{\underset{i\neq j=1}{\sum }}\frac{1}{L_{ij}}. \end{array}$$

#### Network Thresholding and Topological Measures

Appropriate network thresholding is needed to ensure fair comparison of network architecture across different participants and cost thresholds (Bernhardt et al., 2011; Wen et al., 2011; Zhang Z. et al., 2011). The cost threshold value is defined as the ratio between the number of edges in the network and all the possible edges. We evaluated the connectome properties across a wide range of cost threshold (0.01 ≤ cost ≤ 0.40, step = 0.01) (Gong et al., 2009). For SC, we adopted the range of cost thresholds (0.10 ≤ cost ≤ 0.35, step = 0.01) based on the following criteria: (1) each brain network was sparse but fully connected, (2) the average number of connections per node was larger than the log of the number of nodes (Watts and Strogatz, 1998), and (3) the small-worldness of brain networks was >1.2 (Wu et al., 2013). For FC, we adopted the range of cost thresholds (0.20 ≤ cost ≤ 0.31, step = 0.01) based on the following criteria: (1) 80% of nodes were fully connected in 95% of participants (Bassett et al., 2008), (2) the average number of connections per node was larger than the log of the number of nodes (Watts and Strogatz, 1998), and (3) the small-worldness of brain networks was >1 (Watts and Strogatz, 1998; Liu et al., 2008; Wang L. et al., 2009).

We termed the graph theoretical measures obtained at each cost threshold the original graph theoretical measures. The composite graph theoretical measures were then obtained by taking the integral of each original measure (global-wise or nodal-wise) across the selected cost range (Gong et al., 2009).

#### Structural-functional Connectivity Correlation

To examine the relationship between SC and FC, we calculated the Pearson's correlations between the SC and FC, constrained on non-zero edges in the SC (Honey et al., 2009; Hagmann et al., 2010). The analysis was performed on both group-level and individual-level. At the group-level, we obtained the mean SC matrix as the average of all subjects' normalized SC matrix within each group. Similarly, the mean FC matrix was computed as the average of all subjects' raw FC matrix within each group. The non-zero SC network edges were then extracted and correlated with their functional counterparts. This resulted in one SC-FC correlation coefficient for each group. At the individual-level, similar correlation was performed between the strength of the non-zero SC edges and corresponding FC to quantity a single SC-FC “coupling” value for each participant.

### Statistical Analyses

#### Group Differences in Demographic and Clinical Characteristics

One-way analysis of variance (ANOVA) of the three groups followed by *post-hoc* analyses adjusted for all pairwise comparisons (Bonferroni for items with homoscedasticity and Tamhane's T2 for items with heteroscedasticity) was used to compare group differences in demographic and clinical characteristics.

#### Group Differences in Brain Network Global-wise and Nodal-wise Topological Metrics

To examine the group differences in global and local efficiency of the SC and FC, we applied general linear models (GLMs) on the composite graph theoretical measures controlling for age, gender, and handedness. Results were reported at the significance level of *p* < 0.05 for these two global-wise measures.

To examine the group differences in nodal degree centrality and nodal efficiency of the SC and FC, we applied separate GLMs on each composite nodal-wise metric, controlling for age, gender, and handedness (*p* < 0.01, uncorrected).

#### Structural-functional Connectivity Correlation

To determine whether the SC-FC coupling varied between groups, we compared group differences in SC-FC correlations obtained at the group-level (i.e., averaged SC correlation with averaged FC) and individual-level. At the group level, we compared the correlation coefficients between groups using Fisher's z test. At the individual level, we applied GLMs to compare the SC-FC correlation values across groups, controlled for age, gender, and handedness (*p* < 0.05).

#### Cognitive Associations With Global-wise Topology and Structural-functional Correlation

For the composite global-wise SC and FC metrics and the individual SC-FC correlation values, we applied GLMs to examine the associations between z-scores of each cognitive domain and the network measures across all subjects, controlling for age, gender, and handedness (thresholded at *p* < 0.05, Bonferroni corrected for multiple comparisons of seven cognitive domains).

For all ANOVAs and GLMs, we checked the assumptions of variance homogeneity and the normal distribution of the model residuals. There was no substantial violation in normality (Blanca et al., 2017) and homogeneity of variance [(Baguley, 2012) also see section Group Differences in Demographic and Clinical Characteristics] to challenge the robustness of our results.

## Results

### Group Differences in Brain Network Global-wise Metrics

**Figure 2:**
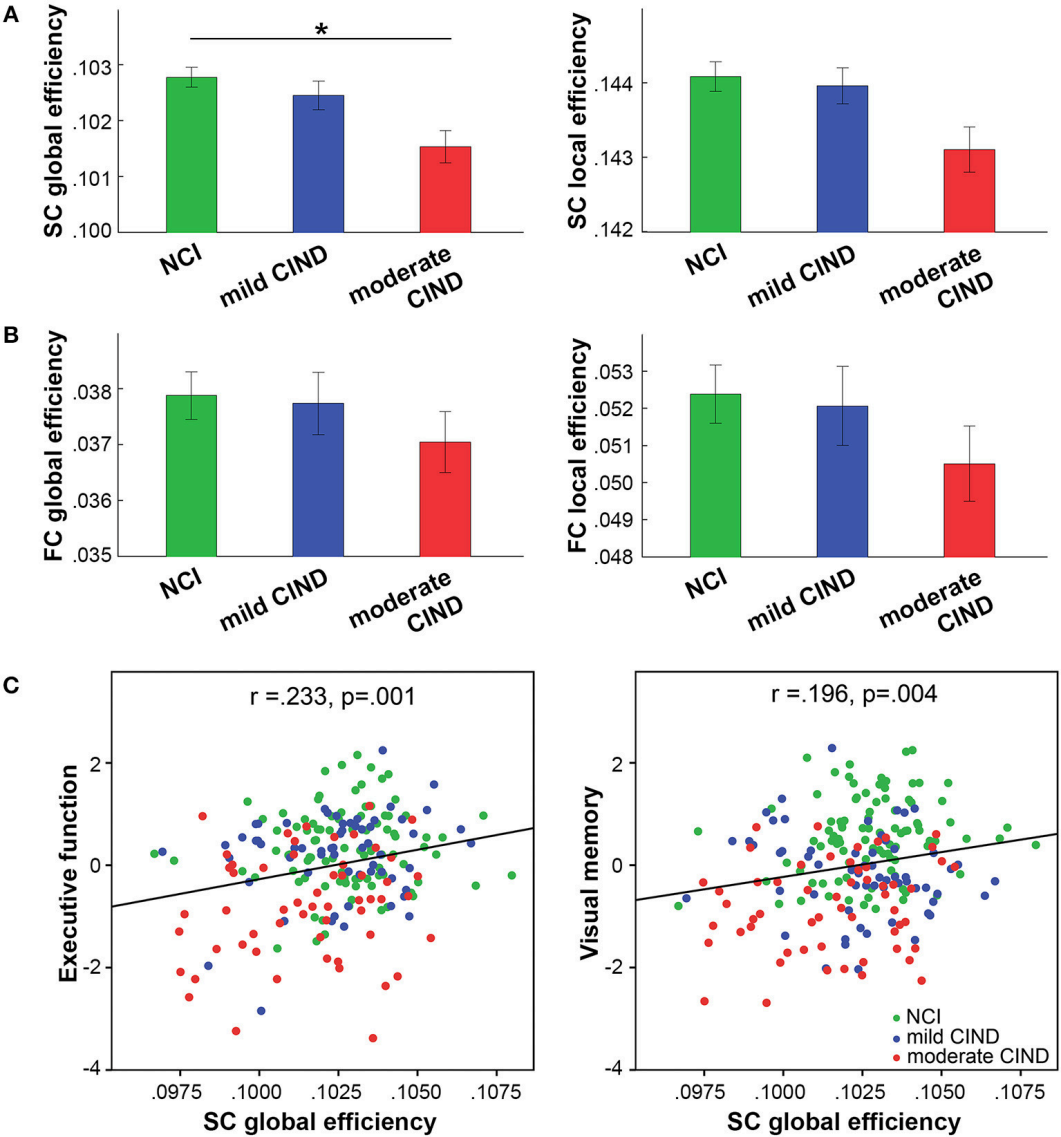
Participants with moderate CIND had reduced network efficiency in brain structural connectome (SC). **(A)** In SC, participants with moderate CIND (but not mild CIND) showed significant reduction in global but not local efficiency compared to participants with no cognitive impairment (NCI) (*p* < 0.05, marked by ^*^). **(B)** In contrast, there was no group difference in functional connectome (FC) global-wise metrics across groups. Error bars represent standard errors within each group. **(C)** Greater structural global efficiency was related to better executive function and visual memory performance (presented as standard residual z-scores controlled for age, gender, and handedness) (*p* < 0.05, multiple comparison corrected).

As hypothesized, we found reduced composite global efficiency of the structural connectome in moderate CIND compared to NCI (*t* = −2.52, *p* = 0.013) (Figures 2A, S3A). Structural global-wise metrics did not differ between mild CIND and NCI nor between mild CIND and moderate CIND.

Interestingly, no significant difference in functional global and local efficiency was observed between mild CIND, moderate CIND, and NCI groups, although we noted that the moderate CIND group showed the trend of lower functional efficiency compared to the other two groups (Figures 2B, S3B).

### Associations Between Global-wise Metrics and Cognitive Performance

Higher structural global efficiency correlated with better executive function (*t* = 3.61, *p* = 0.00038) and visual memory (*t* = 3.02, *p* = 0.003) across all subjects (*p* < 0.05 corrected for seven domains). There was no significant correlation between functional global-wise metrics and cognition. These correlations remained largely unchanged when restricted to the CIND participants (Figure S1).

### Group Differences in Brain Network Nodal-wise Metrics

**Figure 3:**
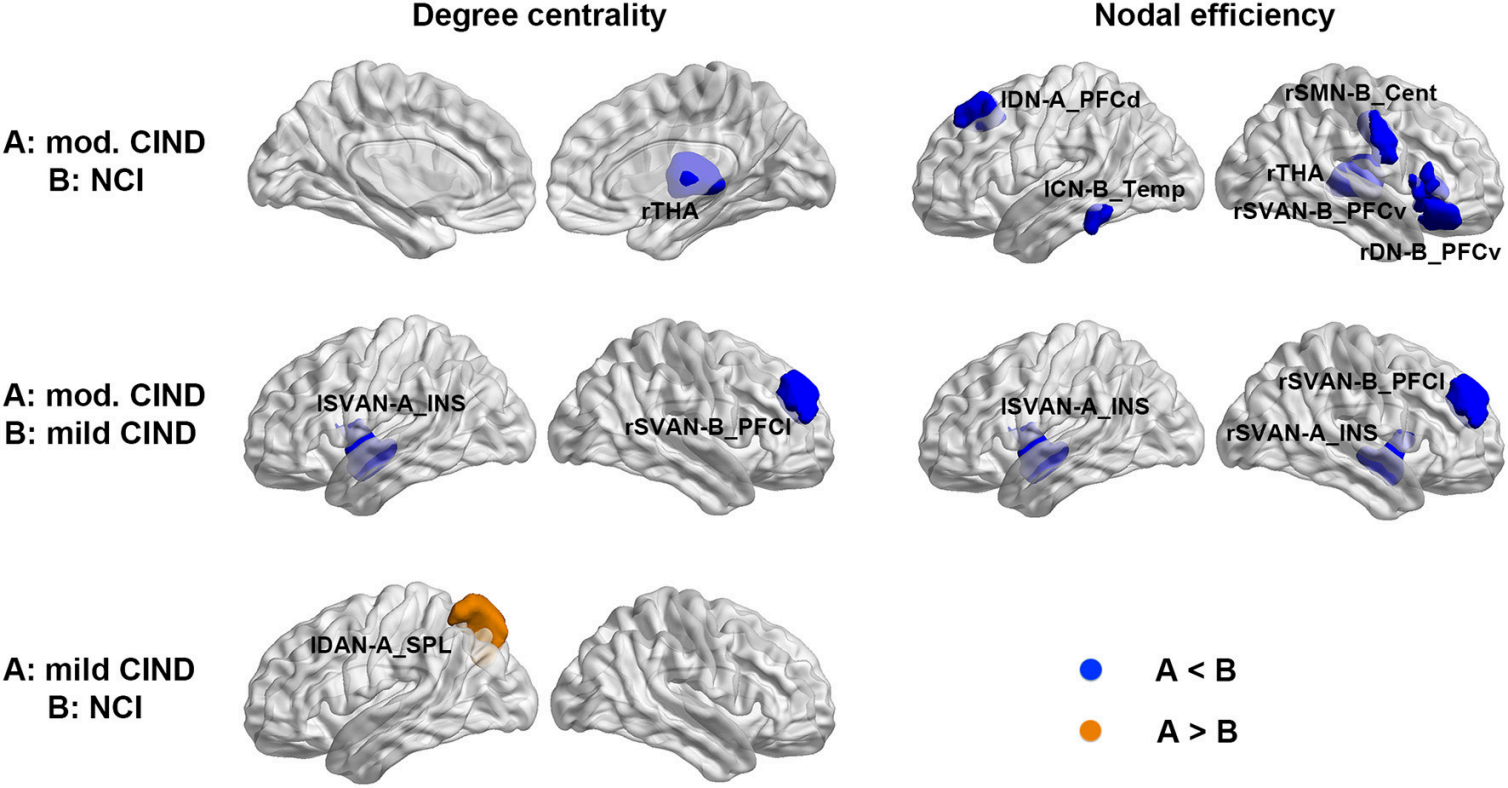
Differential deterioration of structural connectome network topology in mild and moderate CIND. Top: Compared to NCI, moderate CIND group had reduced nodal degree centrality and efficiency in the DN, SN, SMN, and CN as well as the thalamus. Middle: Compared to mild CIND, moderate CIND had reduced nodal degree centrality and efficiency in the salience network. Bottom: Compared to NCI, mild CIND showed increased nodal degree centrality in the dorsal attention network. At each row, compared to group B, increase in group A is highlighted in orange color and decrease in group A is highlighted in blue color (*p* < 0.01). Brain networks were visualized with the BrainNet Viewer (Xia et al., 2013). mod., moderate; THA, thalamus; DN-A_PFCd, default mode network part A, prefrontal cortex dorsal; CN-B_Temp, control network part B, temporal region; SMN-B_Cent, somatomotor network part B, central; SVAN-B_PFCv, salience/ventral attention network part B, prefrontal cortex ventral; DN-B_PFCv, default mode network part B, prefrontal cortex ventral; SVAN-A_INS, salience/ventral attention network part A, insula; SVAN-B_PFCl, salience/ventral attention network part B, prefrontal cortex lateral; DAN-A_SPL, dorsal attention network part A, superior parietal lobule; l, left; r, right.

Consistent with the global-wise SC results, we found nodal-wise structural differences among the three groups. Compared to NCI, moderate CIND had reduced degree centrality in the right thalamus and decreased nodal efficiency in the thalamic, as well as brain regions in DN, control network (CN), somatomotor network (SMN), and Salience/Ventral attention network (SVAN) (Figure 3 top row, Supplementary Table 1). Similarly, compared to mild CIND, moderate CIND showed decreased structural nodal degree centrality and nodal efficiency in the SVAN regions (Figure 3 middle row, Supplementary Table 1). In contrast, compared to NCI, mild CIND had no reductions but only increased degree centrality in the superior parietal lobule of the left dorsal attention network (Figure 3 bottom row, Supplementary Table 1).

**Figure 4:**
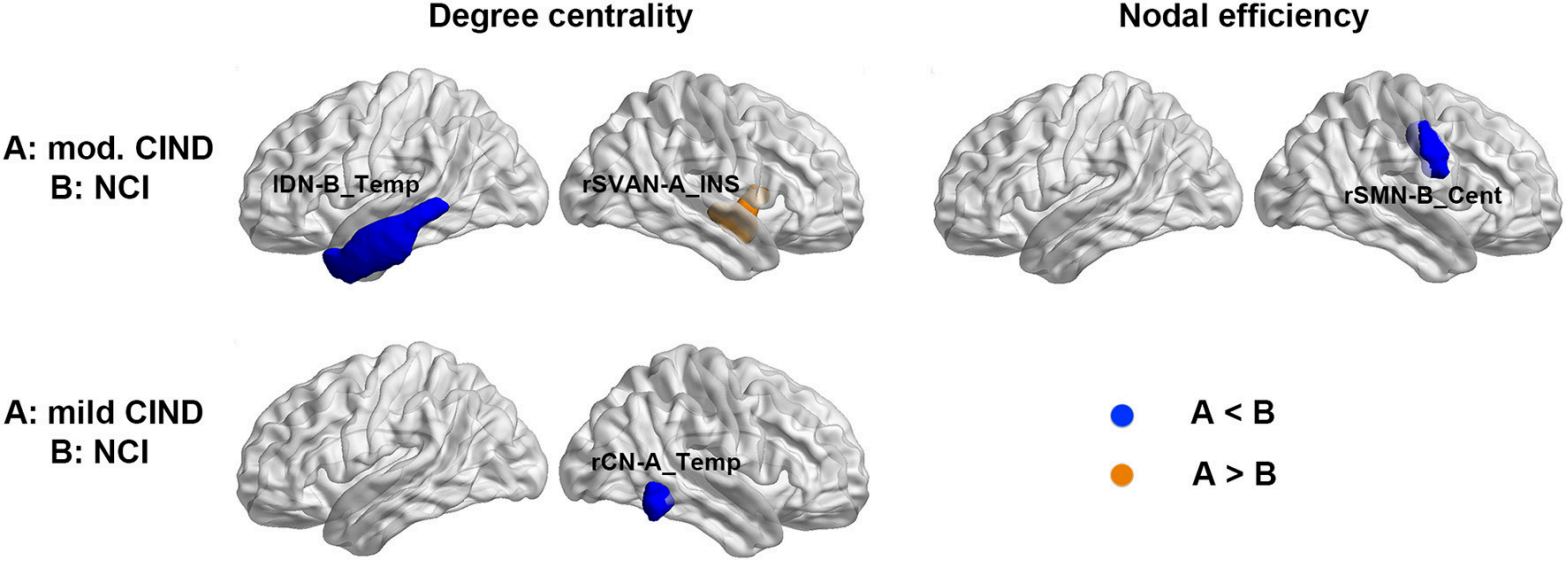
Distinct functional connectome network topological changes in participants with mild and moderate CIND. Top: Compared to NCI, moderate CIND group had reduced nodal degree centrality and efficiency in the default-mode, salience, and somatomotor networks. Bottom: Compared to NCI, mild CIND group had reduced nodal degree centrality in the control network. At each row, compared to group B, a significant increase in group A is highlighted in orange and a significant decrease in group A is highlighted in blue (*p* < 0.01). There was no significant difference in nodal-wise metrics between moderate CIND and mild CIND. DN-B_Temp, default mode network part B, temporal region; SVAN-A_INS, salience/ventral attention network part A, insula; SMN-B_Cent, somatomotor network part B, central; CN-A_Temp, control network part A, temporal region.

Despite the lack of significant difference in the functional global-wise metrics, we found group differences in nodal-wise metrics of the FC. Compared to NCI, moderate CIND had reduced degree centrality at the left temporal region of the DN as well as increased nodal efficiency in the right insula (Figure 4 top row, Supplementary Table 1). Compared to NCI, mild CIND group had reduced degree centrality at the temporal region of CN (Figure 4 bottom row, Supplementary Table 1).

### Group Differences in SC-FC Correlation

**Figure 5:**
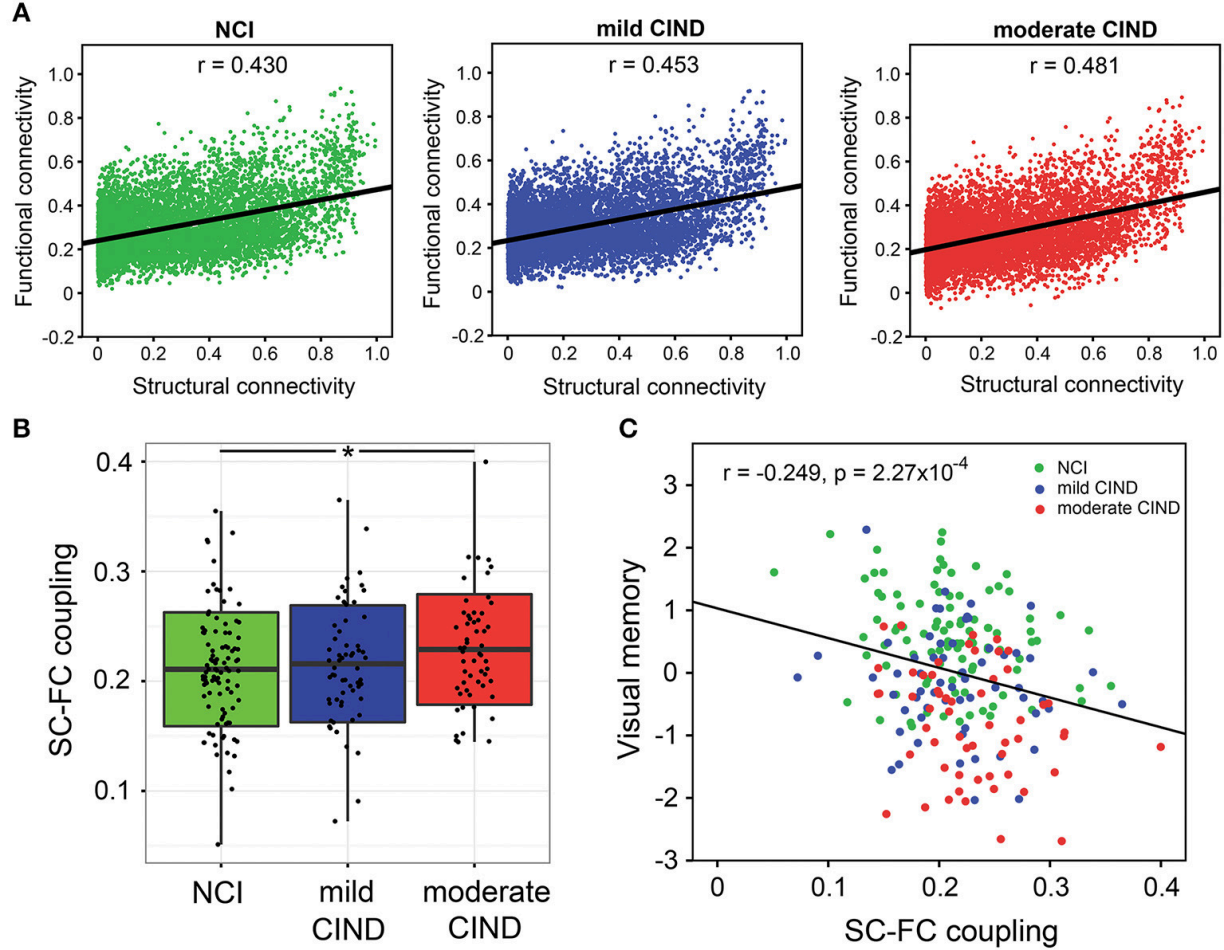
Stronger structural-functional coupling in moderate CIND compared to controls related with poorer cognitive performance. **(A)** Group-level coupling: For each of the three groups (NCI, mild CIND, and moderate CIND), correlations between the functional connectomic (FC) and structural connectomic (SC) strengths across all edges (constrained by non-zero SC edges only) were calculated based on the group-level mean connectivity matrix between the 126 regions of interests. All groups showed significant SC-FC coupling across brain connections. Interestingly, the coupling strength was higher in moderate CIND than NCI while mild CIND was intermediate between the two. **(B)** Individual-level coupling: For each participant, we calculated the SC-FC coupling strength across all brain connections (constrained by non-zeros SC edges only). Moderate CIND (but not mild CIND) showed stronger SC-FC coupling than NCI (denoted by ^*^p < 0.05). **(C)** Across all participants, higher SC-FC coupling was related to poorer visual memory (represented in standard residual z-scores, controlling for age, gender, and handedness, i.e., partial correlation) (*p* < 0.05, with Bonferroni correction for cognitive domains). There was also a trend for executive function and visuoconstruction (*p* < 0.05).

At the group-level, group-averaged FC was closely associated with group-averaged SC across all structurally-defined connections within each group (Figure 5A). Importantly, the moderate CIND group had stronger SC-FC coupling than NCI, with the mild CIND group showing intermediate strength (NCI: *r* = 0.430, mild CIND: *r* = 0.453, and moderate CIND: *r* = 0.481; NCI vs. mild CIND: *p* = 0.0866, NCI vs. moderate CIND: *p* = 0.0246, mild CIND vs. moderate CIND: *p* < 0.001, uncorrected).

At the individual-level, moderate CIND had higher SC-FC correlation than HC (*t* = 2.21,*p* = 0.028) (Figure 5B). Mild CIND exhibited a trend of stronger SC-FC correlation than HC, although the difference did not reach statistical significance.

### Associations Between SC-FC Correlation and Cognitive Performance

Higher SC-FC correlation was associated with poorer visual memory performance across all participants (*t* = −3.764, *p* = 2.18 × 10^−4^) (Figure 5C). The association was significant within NCI (*t* = −2.336, *p* = 0.022) and mild CIND (*t* = −1.912, *p* = 0.061), but not moderate CIND (*t* = −1.645, *p* = 0.106). Similarly, the executive and visuoconstruction showed negative association with SC-FC correlation (executive: *t* = −2.263, *p* = 0.025; visuoconstruction: *t* = −2.434, *p* = 0.016) although they did not survive Bonferroni correction. These correlations remained largely unchanged when restricted to the CIND participants (Figure S2).

## Discussion

Our findings demonstrated differentially disrupted network topology in structural and functional brain connectomes in mild and moderate CIND compared to controls. Specifically, SC global-wise metrics (e.g., global efficiency) but not FC global-wise metrics was reduced in moderate CIND compared to NCI. Importantly, greater SC global efficiency was related to better executive function and visual memory performance. At nodal level, moderate CIND group had reduced structural nodal centrality and efficiency in the thalamus and the DN, SVAN, CN, and SMN but mild CIND group only had increased degree centrality in dorsal parietal regions. Less extensive functional reductions of nodal centrality and efficiency were found in temporal, somatomotor and insula regions in CIND. Furthermore, moderate CIND had increased SC-FC correlation in structurally-defined connections at both group and individual-level relative to controls and mild CIND, indicating more restricted brain functional signals fluctuation by the underlying structural framework. Higher SC-FC correlation was related to poorer verbal memory performance, and with executive and visuoconstruction function to a lesser extent. Our findings provide new insights into the interrelated structural-functional mechanisms underlying large-scale brain network deterioration in different stages of cognitive impairment.

### Topological Changes of Structural Brain Network in CIND and Association With Cognitive Impairment

Disrupted topological organization of brain networks affects the integration of information propagated among distant brain regions (Delbeuck et al., 2003; Dai and He, 2014). Consistent with previous work on amnestic MCI and AD (He et al., 2008; Yao et al., 2010), we found reduced global efficiency of the SC in moderate CIND compared to NCI. In addition, the structural global efficiency of the mild CIND group was intermediate between NCI and moderate CIND, supporting a correspondence between global structural efficiency and CIND progression.

Bolstering the SC global-wise results, we observed altered nodal-wise metrics in the thalamus, frontal and temporal lobe covering the DN, SVAN, CN, and SMN in moderate CIND compared to NCI. The spatial distribution of these brain areas was similar to one recent DTI study of AD showing pattern of thalamic degeneration (Zarei et al., 2010). The thalamus is a crucial brain area that processes and integrates neural activity from widespread neocortical circuits (Postuma and Dagher, 2006) to coordinate information and facilitate communication (e.g., attention, memory, and perception) (Mitchell et al., 2014). The prefrontal regions are thought to be involved in attention and executive functions (Arnsten and Rubia, 2012), while the temporal lobe is associated with semantic memory, visual perception and integrating information from different senses (Dupont, 2002; Olson et al., 2007). Impaired connection between the thalamus and the temporal lobes has been reported to be related to impaired working/short-term memory in AD (Kensinger and Corkin, 2003; Huntley et al., 2011). More importantly, the insula, an integral hub region in the SVAN, plays a critical and causal role in switching between the DN and the CN known to demonstrate competitive interactions during cognitive information processing, and thus facilitates their coordination (Menon and Uddin, 2010; Ng et al., 2016). Combining with association between lower SC global efficiency and poorer performance in executive function and visual memory, our findings suggest that the decreased nodal degree centrality and nodal efficiency in the white matter pathways of these major higher-level cognitive networks and cortico-thalamical circuits could influence information transmission and integration especially in moderate CIND patients.

### Topological Changes of Functional Brain Network in CIND

In contrast to the global structural findings, patients with CIND may have more subtle changes in the global topological organization of the functional brain network. This could be due to our focus on relatively early stages in cognitive impairment, during which disruption has initiated from specific regions and not yet propagated. Alternatively, FC is more robust and resilient against pathological attacks than SC (Vega-Pons et al., 2016), which might be less vulnerable and may even serve as a compensative mechanism for reduced SC in face of early cognitive decline (Caeyenberghs et al., 2013).

Compared to the NCI individuals, patients with CIND showed reduced degree centrality and efficiency at temporal regions. This is consistent with previous findings that the temporal lobe, a site of early AD pathology, exhibits early disconnections with other cortical regions, especially the DN regions, in prodromal AD stages (Sperling et al., 2010; Das et al., 2013; Farras-Permanyer et al., 2015). Furthermore, consistent with the SC results, moderate CIND patients also showed decreased functional nodal efficiency at the somatomotor regions. Accumulating evidence suggests that SC constrains patterns of FC (Honey et al., 2009; McKinnon et al., 2017). We suspect that altered SMN functional deficits might be driven by the altered SMN structural integration. One longitudinal MCI study also found that abnormal intra-SMN functional connectivity and inter-network connectivity between the SMN and the DN could facilitate the disease progression to AD (Zhan et al., 2016). Our results might be interpreted as an aberrant structural and functional communication between the somatomotor and other regions, which parallels previous literature that SMN dysfunctions could represent as an early sign of AD [for review, see (Albers et al., 2015)].

Interestingly, reduced structural integrity in insula was paralleled by enhanced functional degree centrality in insula in CIND. Previous single modality studies have frequently reported SVAN hub connectivity enhancement in the presymptomatic stage (i.e., healthy apolipoprotein-E (APOE) e4 carriers) (Han and Bondi, 2008; Machulda et al., 2011), the amnestic MCI stage (Bai et al., 2012a) and the clinical AD stage (Zhou et al., 2010; Zhou and Seeley, 2014), which represents the disruption of the balance between SVAN and other networks. It has been suggested that SVAN enhancement might be associated with heightened social-emotional sensitivity in AD and correspond to the reciprocal relationship between the DN and the SVAN (Zhou et al., 2010).

Indeed, such opposite pattern in FC and SC is ubiquitous in neurodegenerative diseases. For instance, in patients with amyotrophic lateral sclerosis, Douaud et al. found that FC was increased in regions with decreased SC (Douaud et al., 2011). In preclinical dementia, this FC-SC alteration would be consistent with the current conceptualization of pathology “cascade” (Jack et al., 2013) and might be related to events, such as amyloid deposition (Schultz et al., 2017). Such FC “hyperconnectivity” (Hillary and Grafman, 2017) could either reflect a beneficial compensatory process or an undesirable changes in the neuronal circuits (Verstraete et al., 2010; Schulthess et al., 2016). While our analyses could not directly address the hyperconnectivity hypothesis, our lack of group differences in global FC topology might be partly attributable to related mechanism, contrasting the SC disruption.

### Alterations of SC-FC Coupling in Moderate CIND

Large-scale network functional connectivity is constrained by the underlying anatomical white matter pathways of the human brain (Bullmore and Sporns, 2009; Honey et al., 2009), termed structure-function coupling by some researchers (Zhang Z. et al., 2011). This coupling is partially supported by the positive SC-FC correlation here. Previous evidence suggests altered SC-FC coupling under different physiological (Honey et al., 2009; Hagmann et al., 2010) or pathological states (Skudlarski et al., 2010). To our knowledge, we are the first study demonstrating increased SC-FC coupling in structurally-defined connections in moderate CIND compared to NCI and close association between greater SC-FC coupling and visual memory impairment. While structural organization does constrain functional architecture, there is no one-to-one mapping (Honey et al., 2009; Misic et al., 2016); instead, diverse and highly dynamic properties of large-scale coherent functional network patterns can emerge from the structural topology (Shen et al., 2015), making the interpretation of an increased or decreased coupling in disease complex. On one hand, increased coupling has been reported in epilepsy (Zhang Z. et al., 2011) and schizophrenia (Skudlarski et al., 2010; Cocchi et al., 2014); on the other hand, one migraine study showed decreased coupling, suggesting that changes in SC-FC coupling may be disease-dependent. Similarly, marques under anesthesia were less conscious (less cognitively enabling) and had higher FC-SC similarity (Barttfeld et al., 2015). The undesirable implication of SC-FC alteration in prodromal AD was reflected in the negative correlation between cognitive performance (e.g., visual memory, executive function, and visuoconstruction) and SC-FC coupling. With the more reliably detected alterations in structural connectivity in CIND patients, we argue that the disruption of optimal structural organization may have given rise to SC-FC coupling alteration. Specifically, the degree of structural connectivity integrity might reflect the capacity of the cerebral cortex to maintain functional organization diversity or neural activity interaction (Zimmermann et al., 2016). Therefore, the reduced structural integrity observed in CIND patients may indicate a more rigid structural network configuration, resulting in reduced functional interaction complexity between brain networks (Vincent et al., 2007; van den Heuvel et al., 2013) and hence stronger statistical SC-FC correlation. While we postulated the impact of degraded SC on functional network interactions based on static resting state connectivity measures, future work using time-resolved methods to capture more transient brain functional dynamics would provide further insights about the functional significance of such SC-FC coupling in healthy and diseased populations (Medaglia et al., 2018a). In conclusion, such observations highlight the need to consider multimodal brain connectomes simultaneously to understand the early stage disease pathophysiology.

### Limitations and Future Work

Some limitations apply to the current work. First, our study is a cross-sectional study. As such, this limits generalization and inferences about causal or time-varying relationships. Future studies should consider the use of longitudinal designs which are able to track individual changes in graph topology and neuropsychological performance with time. Second, the examination of brain connectomes in healthy and diseased conditions might be impacted by the choice of brain parcellation and the resolution of the brain parcels. Although generally consistent results could be found regardless of the resolution of the brain parcels (Abou Elseoud et al., 2011; Shehzad et al., 2014), future work is needed to examine the brain structural-functional coupling based on individualized parcellations. Third, there was a lack of multiple comparison correction for the number of brain regions when examining group differences in nodal-wise graph theoretical measures, although a more conservative statistical threshold was used. These results should be treated as exploratory (Amrhein et al., 2017) and worth further investigation. Finally, we showed that SC-FC coupling was progressively increased with greater severity of cognitive deficits, thereby suggesting that CIND individuals might have constrained brain network dynamics. Given that brain network dynamics and their state transition properties have been associated with cognitive performance (Medaglia et al., 2018b) as well as severity of developmental disorders (Watanabe and Rees, 2017), it would be of further interest to investigate disease-related changes in these dynamic properties and their relation to cognitive decline.

## Conclusion

In summary, we documented alterations in brain structural (SC) and functional connectome (FC) of mild CIND and moderate CIND participants compared to NCI individuals using graph theoretical approach. Compared to NCI, CIND individuals showed more disrupted SC at both global and region-specific levels that were associated with impaired executive function and visual memory, while disruption in the FC was more region-specific. Importantly, we found that increased SC-FC coupling was progressively increased from controls to mild and then moderate CIND, and was associated with cognitive impairment. These results suggest that CIND individuals may suffer from constrained brain network dynamics that contribute to poorer cognition. Our findings highlight the importance of using multimodal brain connectome to understand the disease spectrum. Future work is needed to develop and establish the potential value of these measures in a longitudinal context for prognosis and diagnosis of neurodegenerative diseases.

## Author Contributions

JZ, CC, and MI conceived of the study and wrote the manuscript. JZ, JW, RK, KN, and ZH designed the study and wrote the manuscript. JZ, JW, RK, KN, ZH, JC, YW, C-YC, and SH analyzed the data. NV, TW, CC, and MI provided access to patients. All authors contributed to the discussion and result interpretation, read, and approved the final manuscript.

### Conflict of Interest Statement

The authors declare that the research was conducted in the absence of any commercial or financial relationships that could be construed as a potential conflict of interest.
