# Supplementary material for: Alterations in Brain Network Topology and Structural-Functional Connectome Coupling Relate to Cognitive Impairment
Source: Front Aging Neurosci. 2018 Dec 13;10:404. doi: 10.3389/fnagi.2018.00404 (PMC6300727; doi:10.3389/fnagi.2018.00404)
Supplement: Supplementary file 1 [file Data_Sheet_1.pdf]

SUPPLEMENTARY MATERIALS

Supplementary Methods

Graph theoretical analyses

The weighted clustering coefficient of a node i represents the fraction of the node's neighbors that are also neighbors of each other. It is defined as:

$$C_i^w = \frac{\sum_{j,h\in N} \left(w_{ij} w_{jh} w_{ih}\right)^{1/3}}{k_i \left(k_i - 1\right)},$$

where N is the number of nodes and K_i is the degree of node i. Subsequently, the network weighted clustering coefficient is defined as the average of all nodes' clustering coefficient (Saramaki et al., 2007):

$$C_{net}^w = \frac{1}{N} \sum_{i=1}^{N} C_i^w .$$

The path length between nodes i and j was defined as the sum of the edge lengths, which is computed as $1/w_{ij}$, along the path connecting the nodes to each other without passing the other nodes more than once. Then, the shortest path length between nodes i and j, L_{ij} was calculated as the path with the shortest length between the two nodes. The weighted characteristic path length of network, L_{net}^w, quantifies the ability for information propagation in parallel. It was measured by a harmonic mean length between pairs (Watts and Strogatz, 1998; Newman, 2003), to overcome the problem of possibly disconnected network components. Formally, L_{net}^w is computed as follows:

$$L_{net}^w = \frac{1}{\frac{1}{N\left(N-1\right)} \sum_{i=1}^{N} \sum_{i\neq j=1}^{N} \frac{1}{L_{ij}}}.$$

Supplementary Table 1. Brain regions (ROI 126 parcellation) with significant group differences in structural and functional brain connectome nodal-wise metrics. For each of 126 brain regions defined from a previous study (Yeo et al., 2011), we performed multiple linear regression to do pairwise comparisons for each nodal-wise metric, controlling for age, gender and handedness. Uncorrected $p < 0.01$ was considered indicative of a significant difference. Data in parentheses are p values. SC = structural connectome; FC = functional connectome; DN-A_PFCd = Default network part A, prefrontal cortex dorsal; CN-B_Temp = Control network part B, temporal region; SMN-B_Cent = Somatomotor network part B, central; SVAN-B_PFCv = Salience ventral attention network part B, prefrontal cortex ventral; DN-B_PFCv = Default network part B, prefrontal cortex ventral; SVAN-A_INS = Salience ventral attention network part A, insula; SVAN-B_PFCl = Salience ventral attention network part B, prefrontal cortex lateral; DAN-A_SPL = Dorsal attention network part A, superior parietal lobule); DN-B_Temp = Default network part B, temporal region; SVAN-A_INS = Salience ventral attention network part A, insula; SMN-B_Cent = Somatomotor network part B, central; CN-A_Temp = Control network part A, temporal region; L = left hemisphere and R = right hemisphere.

	ROI	T stats of pairwise comparisons		
		**NCI vs mild CIND**	**NCI vs moderate CIND**	**Mild CIND vs moderate CIND**
**SC degree centrality**	R Thalamus	NS	3.10(.002)	NS
	L SVAN-A_INS	NS	NS	2.88(.005)
	R SVAN-B_PFCl	NS	NS	2.74(.007)
	L DAN-A_SPL	-2.63(.009)	NS	NS
**SC nodal efficiency**	R Thalamus	NS	3.53(.0005)	NS
	R SMN-B_Cent	NS	3.11(.002)	NS
	R DN-B_PFCv	NS	3.04(.003)	NS
	L DN-A_PFCd	NS	2.76(.006)	NS
	R SVAN-B_PFCv	NS	2.76(.006)	NS
	L CN-B_Temp	NS	2.74(.007)	NS
	R SVAN-B_PFCl	NS	NS	3.41(.0009)
	L SVAN-A_INS	NS	NS	3.08(.003)
	R SVAN-A_INS	NS	NS	2.81(.006)
**FC degree centrality**	L DN-B_Temp	NS	3.22(.002)	NS
	R SVAN-A_INS	NS	-2.71(.007)	NS
	R CN-A_Temp	2.82(.005)	NS	NS
**FC nodal efficiency**	R SMN-B_Cent	NS	2.65(.009)	NS

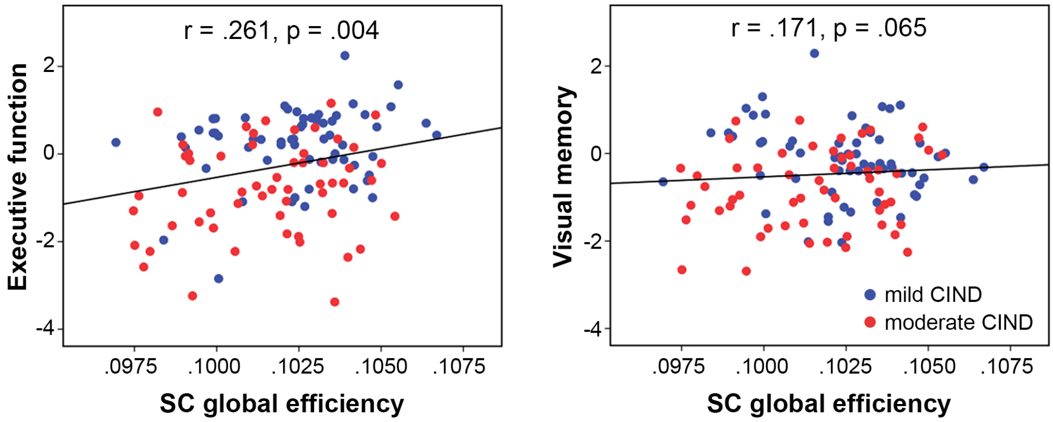

Figure S1. Greater structural global efficiency was related to better executive function and visual memory performance in CIND patients. The cognitive performance was presented as standard residual z-scores controlled for age, gender, and handedness.

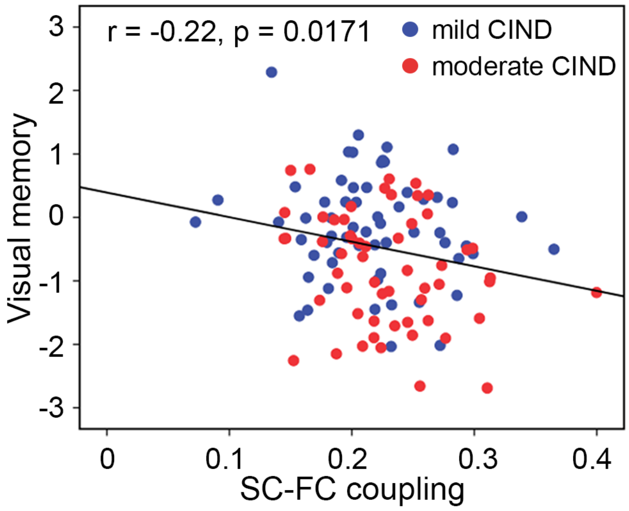

Figure S2. Higher SC-FC coupling was related to poorer visual memory in CIND patients. The cognitive performance was presented as standard residual z-scores controlled for age, gender, and handedness.

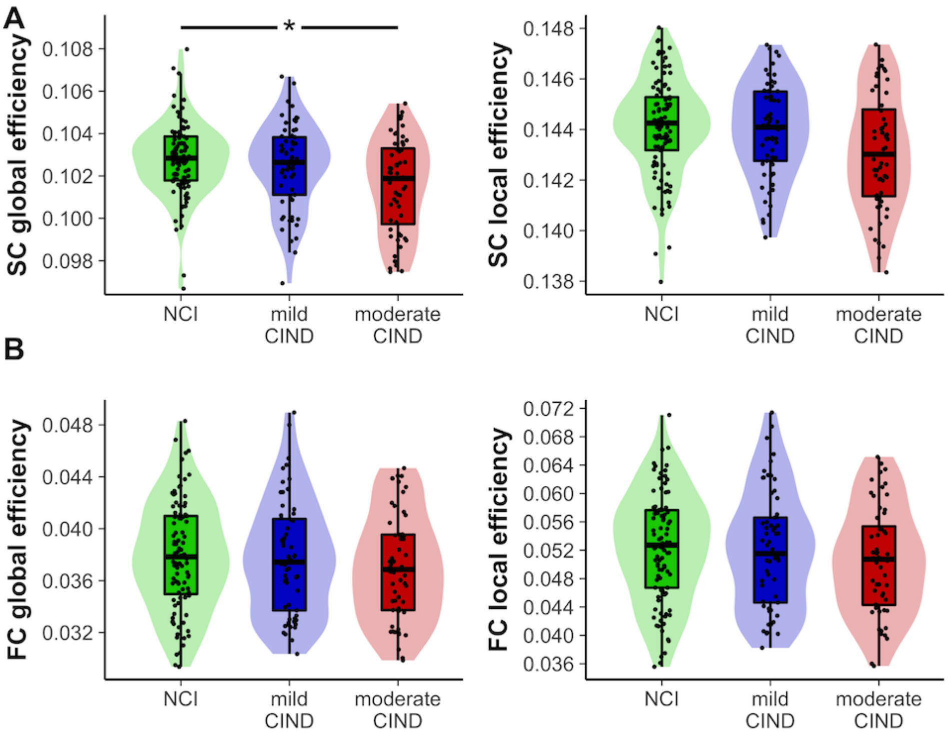

Figure S3. Participants with moderate CIND had reduced network efficiency in brain structural connectome (SC). **(A)** In SC, participants with moderate CIND (but not mild CIND) showed significant reduction in global but not local efficiency compared to participants with no cognitive impairment (NCI) ($p < 0.05$, marked by *). **(B)** In contrast, there was no group difference in functional connectome (FC) global-wise metrics across groups. Shades represent density of data points of the respective graph metric.

References

Newman, M.E.J. (2003). The structure and function of complex networks. *Siam Review* 45(2)**,** 167-256. doi: Pii S0036144503424804

Doi 10.1137/S003614450342480.

Saramaki, J., Kivela, M., Onnela, J.P., Kaski, K., and Kertesz, J. (2007). Generalizations of the clustering coefficient to weighted complex networks. *Phys Rev E Stat Nonlin Soft Matter Phys* 75(2 Pt 2)**,** 027105. doi: 10.1103/PhysRevE.75.027105.

Watts, D.J., and Strogatz, S.H. (1998). Collective dynamics of 'small-world' networks. *Nature* 393(6684)**,** 440-442.

Yeo, B.T., Krienen, F.M., Sepulcre, J., Sabuncu, M.R., Lashkari, D., Hollinshead, M., et al. (2011). The organization of the human cerebral cortex estimated by intrinsic functional connectivity. *J Neurophysiol* 106(3)**,** 1125-1165. doi: 10.1152/jn.00338.2011.
